# Response to Austad: Offering a Range of Methods, Including Fertility Awareness Methods, Facilitates Method Choice

**DOI:** 10.9745/GHSP-D-16-00115

**Published:** 2016-06-20

**Authors:** Shawn Malarcher, Madeleine Short Fabic, Jeff Spieler, Ellen H Starbird, Clifton Kenon, Sandra Jordan

**Affiliations:** a United States Agency for International Development, Office of Population and Reproductive Health, Washington, DC, USA; b Independent Consultant, Washington, DC, USA

## Abstract

When selecting a contraceptive method, women and men consider various attributes in addition to effectiveness, such as side effects, return to fertility, level of medical intervention, and interference with sexual activity. Offering a range of methods, including fertility awareness methods that meet the standard to be considered modern, helps to address these considerations, facilitating method choice.

See related article by Austad.

We appreciate the “Taking Exception” article by Austad and colleagues^1^ and their critical review of the important issue of contraceptive classification first introduced in our March 2016 *Global Health: Science and Practice* (GHSP) article, “Fertility Awareness Methods: Distinctive Modern Contraceptives.”^2^ We support the authors’ assertion that contraceptive effectiveness is a crucial concern for family planning program managers and potential contraceptive users. We also agree that, as with all other contraceptives, fertility awareness methods (FAMs) have limitations that make them less appropriate for some women. We firmly disagree, however, with the idea that the contraceptive needs of all women and their partners can be met by a limited set of contraceptives prioritized according to one attribute—effectiveness. Once again, we assert that family planning programming should be based first and foremost on voluntary and informed contraceptive choice. We further argue our position by discussing a few points raised by Austad et al.

## FAMS AS MODERN CONTRACEPTION NOT A NEW IDEA

Austad et al. refer to the concepts put forth in our original GHSP commentary as reclassifying the Standard Days Method (SDM) and Lactational Amenorrhea Method (LAM) from traditional to modern contraceptives. In fact, the United States Agency for International Development (USAID) has never considered these methods traditional. They are scientifically based and tested approaches that draw on traditional practices. LAM and SDM have been classified as modern methods for decades. Indeed, USAID has supported the Demographic and Health Surveys to collect and report data on LAM as a modern method since 1998 and on SDM since SDM programming began being taken to scale in the mid-2000s.

LAM and SDM have been classified as modern methods for decades.

## MODERN/TRADITIONAL DICHOTOMY USED FOR PROGRAM PLANNING AND REPORTING—NOT FOR COUNSELING

We agree with the authors that family planning clients should be given comprehensive and accurate information on contraceptive options, including method effectiveness rates and what those rates mean. We also acknowledge that the ability to provide accurate and comprehensive information remains a serious challenge for many programs. The classification of “modern” and “traditional” is, however, meaningless for the purposes of counseling. Counseling tools such as the World Health Organization’s “Decision-Making Tool”^3^ and the Population Council's “Balanced Counseling Strategy”^4^ make no reference to “modern” and “traditional.” Rather, these terms are used by program managers and decision makers to distinguish those methods that are supported by programs and those contraceptive users who are counted by indicators used for measuring progress toward national and international goals. By arguing that LAM, SDM, and the TwoDay Method (TDM) should be classified as “traditional” methods, Austad and colleagues are, in effect, arguing that these methods should not be supported by organized family planning programs. Recognizing these methods as “modern” increases the likelihood that FAMs will be included in programs, that counseling will include FAMs, and that clients will have the information to decide which method—whether FAMs or another method—can help them meet their fertility intentions.

The modern/traditional contraceptive classification system is useful only for program managers and decision makers—not for the purposes of counseling clients.

## MEDICAL INTERVENTION FACILITATES, BUT NOT REQUIRED FOR, PREGNANCY PREVENTION

Austad and colleagues support the definition for modern contraception put forth by Hubacher and Trussell: “technological advances designed to overcome biology” that “enable couples to have sexual intercourse at any mutually desired time.”^5^ This definition argues that averting pregnancy requires medical intervention, specifically in the form of a “technology.” USAID has supported technological advances in contraceptive development for decades and has contributed to the development of some of the most effective methods on the market, including implants and intrauterine devices (IUDs). At the same time, we recognize that there will always be some women who are unwilling or unable to use devices or drugs. Our work to support the development of scientifically based, effective means to meet the needs of men and women who prefer nonmedical ways of regulating their fertility led to the development of LAM, SDM, and TDM. The proposal by Hubacher and Trussell, and supported by Austad et al., means, in effect, that approaches used by men and women who prefer contraception based on a nonmedical intervention could *never* be recognized as “modern” contraception and, as argued earlier, that these methods would be left out of programming and actively discouraged by providers. These women and men would never be counted as “contraceptive users” by international standards and their choice would never be supported by programming efforts.

## FAMS SHOULD BE OFFERED WITHIN THE CONTEXT OF A BROAD METHOD MIX

All counseling should start by identifying and attempting to meet the needs of the individual client. Studies show that a woman is more likely to continue using a method when she is able to access her preferred method, even if that method is not the most effective method available.^6^ Austad et al. imply that USAID is suggesting prioritizing FAM over other contraceptives. In fact, USAID consistently and resolutely promotes and supports provision of method choice, which includes long-acting reversible contraception, permanent methods, post-coital methods, barrier methods, and fertility awareness methods.

Women are more likely to continue using contraception when they can use their preferred methods, even if those methods are not the most effective ones.

## SIDE EFFECTS ARE REAL AND A COMMON CAUSE OF DISCONTINUATION

Austad and colleagues dismiss women’s concerns about contraceptive side effects noting, “… research shows that the *absolute effectiveness* of a method for pregnancy prevention is *the most important factor* cited by end users when choosing a method, even over other considerations such as side effects.” This statement does not accurately capture the conclusions of the work cited. In Snow et al. (1997),^7^ the conclusions of the evidence review on users’ perspectives on fertility regulation were captured as:

- Contraceptive users lack complete information about both methods and services.
- Women’s and men’s needs and preferences for contraception change over time and vary with the person’s stage of life.
- Universally, women and men would like a method that is safe and effective, but it is not clear what these concepts mean. Side effects and health concerns (particularly with respect to hormonal methods) and method failure (particularly with respect to barrier methods and periodic abstinence) are the major reasons why women discontinue or do not use contraception.
- Individual perspectives and preferences vary widely and defy generalisation.
- The limited range of methods available in many developing countries necessarily limits people’s perceptions and preferences.
- Research on people’s reactions to a hypothetical method does not usually yield information predictive of subsequent use or behaviour with the method.
- There is a particular lack of information about the perspectives of men, adolescents, women having an abortion, especially repeat abortion, and women in the post-partum period.

The influence of method-related side effects experienced by women is real. Castel and Askew (2015) found that 38% of women with an unmet need for modern contraception discontinued using a modern contraceptive in the past.^8^ They also note that one-third of women who start using a modern contraceptive method will stop using it in the first year, and over half before 2 years. Method-related concerns are one of the most common reasons for discontinuation. Women reporting method-related reasons for not using a modern method account for about two-thirds of unmet need in sub-Saharan Africa (67%) and South Central Asia (71%), and for 79% of unmet need in Southeast Asia.^9^

Effectiveness is one important attribute users consider when selecting a contraceptive method. There are, however, many other aspects that women and men prioritize when choosing a contraceptive method, such as side effects, return to fertility, level of medical intervention, and effect on sexual activity (Table). Offering a range of methods, including FAMs, helps to address these considerations, facilitating method choice.

Effectiveness is only one important attribute among many that users consider when selecting a contraceptive method.

**TABLE:** Ways to Classify Commonly Used Contraceptives for Consideration by Countries and Family Planning Programs

Classification System	Female Ster.	Vas.	Implant	Copper IUD	Hormonal IUS	Injectable	OCP	ECP, 1.5 mg LNG	Male Condom	Female Condom	Dia-phragm	LAM	SDM	TDM	Rhythm/Calendar Method	With-drawal
**Modern or traditional**	M	M	M	M	M	M	M	M	M	M	M	M	M	M	T	T
**Level or tier of effectiveness**	1	1	1	1	1	2	2	3^a^	3	3	3	2	3	3	4	4
**Need for programme support**	Hi	Hi	Hi	Hi	Hi	Me	Lo	Lo	Lo	Lo	Lo	Me	Me	Me	No	No
**Duration of labelled use**	P	P	LA	LA	LA	MA	SA	SA	SA	SA	SA	MA	SA	SA	SA	SA
**Male- or female-controlled, or both**	FC	MC	FC	FC	FC	FC	FC	FC	MC	FC	FC	FC	Both	Both	Both	MC
**Coitally dependent/related**	N	N	N	N	N	N	N	Y	Y	Y	Y	N	Y	Y	Y	Y
**Need for surgical procedure to use**	Y	Y	Y	N	N	N	N	N	N	N	N	N	N	N	N	N
**Presence of hormones**	N	N	Y	N	Y	Y	Y	Y	N	N	N	N	N	N	N	N
**Client's ability to discontinue without needing a provider**	N	N	N	N	N	Y	Y	Y	Y	Y	Y	Y	Y	Y	Y	Y
**Return to fertility after discontinuation of method**	U	U	I	I	I	D	I	I	I	I	I	D	I	I	I	I

Abbreviations of contraceptive methods: ECP, emergency contraceptive pill; IUD, intrauterine device; IUS, intrauterine system; LAM, Lactational Amenorrhea Method; LNG, levonorgestrel; OCP, oral contraceptive pill; SDM, Standard Days Method; Ster., sterilization; TDM, TwoDay Method; Vas., vasectomry.

a If 100 women used progestin-only ECPs, one would likely become pregnant (from *Family Planning: A Global Handbook for Providers*, 2011 update). Please note that effectiveness in ECP studies was computed on women’s use after one act of protected intercourse, which is different from analyses of other contraceptive effectiveness studies.

**Legend:**

Modern or traditional: M, modern; T, traditional.

Level or tier of effectiveness: 1, 2, 3, or 4 (as per *Family Planning: A Global Handbook for Providers*, 2011 update)

Need for programme support: Hi, high (requires clinic setting with trained and skilled providers); Me, medium (can be provided in non-clinical setting by trained and skilled providers); Lo, low (can be provided in community distribution programmes, over the counter, or in informal settings); No, none.

Duration of labelled use: P, permanent; LA, long-acting; MA, medium-acting; SA, short-acting. Note: Medium-acting is not a commonly used category but is presented here to distinguish it from the methods which are labelled for a lesser period of effect.

Male- or female-controlled, or both: MC, male-controlled; FC, female-controlled; Both, requires cooperation of both the man and the woman.

Coitally dependent/related (requires specific intervention at the time of intercourse): Y, yes; N, no.

Need for surgical procedure to use: Y, yes; N, no.

Presence of hormones: Y, yes; N, no.

Client's ability to discontinue without reliance on a provider: Y, yes; N, no.

Return to fertility after discontinuation of method: I, immediate; D, delayed; Ne, never; U, uncertain success after reversal.

Source: Developed by the World Health Organization for a consultation on contraceptive classification and presented at the 2016 International Conference on Family Planning; 2016 Jan 25–28; Nusa Dua, Indonesia.

## Notes

**Peer Reviewed**
